# A comparison of patients’ and dietitians’ perceptions of patient‐centred care: A cross‐sectional survey

**DOI:** 10.1111/hex.12868

**Published:** 2019-01-22

**Authors:** Ishtar Sladdin, Lauren Ball, Brigid M. Gillespie, Wendy Chaboyer

**Affiliations:** ^1^ Menzies Health Institute Queensland Griffith University Southport Queensland Australia; ^2^ School of Allied Health Sciences Griffith University Southport Queensland Australia; ^3^ School of Nursing and Midwifery Griffith University Southport Queensland Australia

**Keywords:** comparison, cross‐sectional survey, dietitians, patient‐centred care, patients, perceptions

## Abstract

**Aim:**

The aim of this study was to compare patients’ and dietitians’ perceptions of patient‐centred care (PCC) in dietetic practice.

**Methods:**

Participants were as follows: (a) adult patients who had attended ≥1 individual dietetic consultation with an Accredited Practicing Dietitian (APD) working in primary care; and (b) APDs with experience working in primary care. A cross‐sectional survey was undertaken using a patient‐ and dietitian‐reported inventory to measure PCC in dietetic practice. The inventory comprised of five previously validated scales: The Communication Assessment Tool; the 9‐item Shared Decision‐Making Questionnaire; the Patient‐Doctor Depth of Relationship Scale; the Schmidt Perception of Nursing Care Scale‐Seeing the Individual Patient sub‐scale; and the Person‐Centred Practice Inventory—Staff ‐Providing Holistic Care sub‐scale. Descriptive statistics were used to analyse participant characteristics and to compute total scores for the five scales. The Mann‐Whitney *U* test was used to compare median scores between patients and dietitians.

**Results:**

One‐hundred and thirty‐three patients and 180 dietitians completed the survey. Patients reported significantly higher scores compared to dietitians for “shared decision‐making” (*P *= 0.004), but significantly lower scores for “providing holistic and individualized care” (*P *= 0.005), “knowing the patient/dietitian” (*P* = 0.001) and “caring patient‐dietitian relationships” (*P* =0.009).

**Conclusion:**

This study highlighted potentially important differences between patients’ and dietitians’ perceptions of PCC and identified key aspects of dietetic care requiring practice improvements. Strategies are needed to bridge gaps between dietitians’ and patients’ perceptions and enhance PCC in dietetic practice. These findings suggest that dietitians should focus on individualizing nutrition care, gaining a holistic understanding of their patients and knowing/understanding each patient.

## 1 INTRODUCTION

The concept of patient‐centred care (PCC) has been topical since the 1950s and is widely recognized as an essential component of health care.^1^, ^2^ PCC refers to care that considers patients’ unique values, needs and preferences and is tailored accordingly.^3^, ^4^ A number of dimensions of PCC have been described in the literature, including shared decision‐making; clinician‐patient relationship; personalized/individualized care; providing holistic care; and good clinician‐patient communication.^5^, ^6^, ^7^

Patient‐centred care has been associated with significant benefits for patients, particularly patients with chronic disease.^8^, ^9^, ^10^ Benefits for patients include increased satisfaction, improved quality of life, enhanced engagement in care, improvements in biomedical markers of disease, reduced symptom burden (eg, reduced nausea, improvements in depression) and decreased healthcare utilization.^9^, ^11^, ^12^, ^13^ Patients’ desires for PCC have also been frequently documented.^14^, ^15^, ^16^, ^17^ Further, in the absence of PCC, patients have reported feeling disengaged, uncomfortable and uncertain about care plans, describe challenges with understanding the information provided and perceive care plans as unhelpful and unrealistic.^14^, ^16^, ^18^ Considering the benefits of and patients’ desires for PCC, it is important that healthcare professionals adopt these practices.

Dietitians are trained to support patients to achieve a healthy diet and lifestyle for the prevention and management of chronic disease.^19^ However, findings from a systematic review of randomized controlled trials reported mixed evidence of dietitians being effective at helping patients decrease risk factors of chronic disease.^20^ The review suggested there is room for improvement regarding dietitians’ practices. Since patient‐centred practices by health professionals have been associated with significant benefits for patients in other contexts,^9^, ^11^, ^12^, ^13^ and are clearly valued by patients,^14^, ^15^, ^16^, ^17^ enhanced PCC in dietetics may contribute to dietitians’ effectiveness in this area.

Gaining patients’ feedback regarding their care experiences is essential to making meaningful improvements to health care.^1^, ^21^ However, to effectively change practice, it is important to understand the perspectives of various stakeholders and any differences in their views.^22^ Thus, the aim of this study was to compare patients’ and dietitians’ perceptions of PCC in dietetic consultations. This understanding will help identify any discrepancies between patients’ and dietitians’ perceptions, and aspects of care requiring improvements, to inform the development of strategies that align care with patients’ needs and expectations, which is key to providing PCC.

A conceptual model of PCC in dietetic practice^23^, ^24^ guided the selection of instruments to measure and compare patients’ and dietitians’ perceptions of PCC. The conceptual model comprises five dimensions: (a) patient‐dietitian communication—comprising a set of skills including rapport building, understanding patients’ perspectives and demonstrating empathy, for example; (b) shared decision‐making—an interactive process where both parties contribute equally to the consultation and patients are actively engaged in decision‐making; (c) caring patient‐dietitian relationships—genuine, reciprocal relationships based on trust, respect and understanding; (d) knowing the patient/dietitian—involves dietitians gaining knowledge and understanding of their patients and patients feeling understood by the dietitian; and (e) providing holistic and individualized care—dietitians gain a holistic understanding of the patient and individualize care to patients’ unique needs, values and preferences.

## 2 METHODS

A cross‐sectional survey was undertaken between November 2017 and May 2018. The approval was obtained from the institution's Human Research Ethics Committee (REF: 2017/730).

### 2.1 Sample and setting

Participants were as follows: (a) patients who were ≥18 years, English speaking and had attended at least one individual dietetic consultation with an Accredited Practicing Dietitians (APD) working in primary care in Australia and (b) Australian APDs who self‐reported previous or current experience working as a dietitian in primary care.

Six dietetic practices across Queensland (n = 3), New South Wales (n = 2) and Victoria (n = 1) participated in the recruitment of patients to complete the survey. While some practices employed multiple dietitians, other practices comprised a single dietitian working independently.

### 2.2 Measures

The recently validated patient^23^ and dietitian versions^24^ of an inventory to measure PCC in dietetics were used in this study. The inventory was comprised of five previously validated scales, modified slightly to reflect the dietetic context (eg ‘doctor’ or ‘nursing staff’ replaced with ‘dietitian’).

#### 2.2.1 The Communication Assessment Tool

A 14‐item self‐report measure of patients’ perceptions of their physicians’ communication skills with a five‐point response scale ranging 1 (poor) to 5 (excellent).^25^ Example items include the following: (1) the dietitian greeted me in a way that made me feel comfortable; (2) treated me with respect; and (3) showed interest in my ideas about my health.

#### 2.2.2 The 9‐item Shared Decision‐Making Questionnaire (patient and physician version)

A 9‐item self‐report measure of patients’ and physicians’ perceptions of shared decision‐making, respectively, with a six‐point response scale ranging 0 (completely disagree) to 5 (completely agree).^26^, ^27^ Example items include the following: (1) my dietitian wanted to know exactly how I wanted to be involved in making decisions; (2) told me that there are different options to address my nutrition care; and (3) clearly explained the advantages and disadvantages of different options.

#### 2.2.3 The Patient‐Doctor Depth of Relationship Scale

An 8‐item self‐report measure of patients’ perceptions of the physician‐patient relationship with a five‐point response scale ranging 0 (disagree) to 4 (totally agree).^28^ Example items include the following: (1) I know this dietitian well; (2) this dietitian knows me as a person; and (3) this dietitian really knows how I feel.

#### 2.2.4 The Seeing the Individual Patient sub‐scale (from the Schmidt Perception of Nursing Care Scale)

A 5‐item self‐report measure of patients’ perceptions of nursing staffs’ provision of individualized care with a five‐point response scale ranging 1 (strongly disagree) to 5 (strongly agree).^29^ Example items include the following: (1) the dietitian treated me like a special person; (2) I knew my dietetic care was specifically tailored to my needs; and (3) the dietitian took time to find out more about me as a person.

#### 2.2.5 The Providing Holistic Care sub‐scale (from the Person‐Centred Practice Inventory‐Staff)

A 3‐item self‐report measure or nursing staffs’ perceptions of holistic care with a five‐point response scale ranging 1 (strongly disagree) to 5 (strongly agree).^30^ Example items include the following: (1) my dietitian strives to gain a sense of me as a whole person; (2) assesses my needs, taking account of all aspects of my life; and (3) took time to find out more about me as a person.

For this study, only items that had comparable results for both the patient and dietitian versions during psychometric testing^23^, ^24^ were included in analyses so that accurate comparisons could be made. For example, results from factor analysis revealed that Communication Assessment Tool (CAT) items 6, 11 and 14 loaded strongly on the communication factor in the patient sample but failed to load strongly on any component in the dietitian sample. Therefore, these items were excluded for the purpose of this study. A total of 29 items were included from a potential total of 39 items (for details, refer to Table 1).

**Table 1:** Characteristics of the modified 29‐item inventory used in this study

Label (ie dimension of PCC)	Scale(s) included	No. of items	Factor loadings (range)^a^		Cronbach's *α* b		Inter‐item correlations range c	
			Dietitian (n = 180)	Patient (n = 133)	Dietitian	Patient	Dietitian	Patient
Patient‐dietitian communication	CAT (items 1‐5, 10, 13)	7	0.528‐0.871	0.594‐0.954	0.84	0.94	0.34‐0.61	0.55‐0.84
Shared decision‐making in dietetics	SDM‐Q‐9 (items 2‐9)	8	0.623‐0.888	0.553‐0.984	0.91	0.96	0.44‐0.73	0.61‐0.88
Holistic, individualized dietetic care	SPNCS (items 1‐5) and PCPI‐s (items 1‐3)	8	0.586‐0.967	0.666‐0.897	0.91	0.95	0.42‐0.86	0.57‐0.89
Knowing the patient/dietitian	PDDRS (items 1‐3)	3	0.737‐0.828	0.416‐0.949	0.82	0.87	0.58‐0.62	0.55‐0.86
Caring patient‐dietitian relationships	PDDRS (items 5‐7)	3	0.647‐0.864	0.633‐0.892	0.73	0.91	0.33‐0.56	0.70‐0.82

CAT, Communication Assessment Tool PCC, patient‐centred care; PCPI‐s, Person‐Centred Practice Inventory‐Staff (Providing Holistic Care sub‐scale); PDDRS, Patient‐Doctor Depth of Relationship Scale; SDM‐Q‐9, 9‐item Shared Decision‐Making Questionnaire; SPNCS, Schmidt Perception of Nursing Care Survey (Seeing the Individual Patient sub‐scale).

Criteria: ^a^Loadings ≥0.45 considered significant; ^b^Cronbach's alpha ≥0.70; ^c^Correlations ≥0.3 (with >0.70 indicating potential item redundancy).

### 2.3 Data collection

Dietitians briefly explained the study to consecutive, eligible patients, with a description provided by the research team. Patients who were willing to participate were provided with the research pack at the end of the consultation. The research pack included the inventory, demographic questions, participant information sheet and a reply‐paid envelope. Participants were instructed to complete the survey at a convenient time and return the survey to the research team via the reply‐paid envelope.

Several strategies were employed to distribute the survey to dietitians. The survey was distributed in the form of an e‐survey via the Dietitians Association of Australia (DAA) weekly member email, the Dietitian Connection weekly e‐newsletter and several dietetic specific social media sites. Three reminder emails were distributed via both the DAA member email and Dietitian Connection e‐newsletter during the data collection period. A paper‐based version was also distributed at an annual dietetic seminar (Dietitians Unite). For both patients and dietitians, completion of the survey implied consent.

### 2.4 Data analysis

Data were entered into Statistical Package for the Social Sciences (SPSS) version 24 (IBM, Chicago, IL, USA) and subject to data cleaning. There were no missing data for the dietitian survey, and very few missing data for the patient survey (ranging 0.7%‐3.8% for each item on the inventory and 0.75%‐4.5% for demographic data).

Exploratory factor analysis was performed to evaluate the factor structure of the modified 29‐item inventory, and Cronbach's alpha (criteria ≥0.70) was computed to evaluate the internal consistency of each scale.^31^

Descriptive statistics were analysed using absolute (numbers) and relative (%) frequencies of the total scores for each of the five scales, and to evaluate the distribution of the data (ie, skewness and kurtosis). Using the Shapiro‐Wilk test,^32^ continuous variables (participants’ age, years’ practice experience, hours worked per week and scale scores) were non‐normally distributed and therefore analysed using median and interquartile range (IQR).

Total scores were computed for each scale of the inventory. The Mann‐Whitney *U* test was used to determine whether there were differences in the median scores between patients and dietitians (ie, whether one group had values higher than the other).^33^ Differences between patients and dietitians were considered statistically significant at or below *P* < 0.05.

## 3 RESULTS

Four hundred and forty surveys were distributed to patients and 133 completed and returned the survey, representing a response rate of 30.2%. One‐hundred and eighty dietitians completed the survey. An exact response rate was not possible to determine for dietitians as the survey was distributed anonymously to all Australian APDs who were members of the DAA (>6800 members).^34^ While the survey was not distributed exclusively to dietitians who worked in primary care, only those with experience working in primary care were asked to complete the survey.

Patients’ and dietitians’ median (IQR) age was 61 (23) and 34 (19) years, respectively. Eighty (60.6%) patients and 178 (98.9%) dietitians were female. Most patients reported being born in Australia (85.5%) and the UK (7.6%). Five participants reported being born in Europe (3.8%), and one each in the United States, Canada, China, Africa and New Zealand. Eighty‐six (67.2%) patients reported having a regular dietitian. Dietitians reported a median (IQR) of 8 (18.0) years of experience working as a dietitian and worked a median (IQR) of 27.5 (23) hours per week as a dietitian. Eighty‐two (46.6%) dietitians reported having additional training beyond graduation; open responses indicated additional training included a certificate or diploma (eg, paediatric nutrition, diabetes education, counselling, sports nutrition, business management); courses or workshops (eg, motivational interviewing, FODMAPs, non‐diet approach, cognitive behavioural therapy); and/or an Honours, Masters or Doctorate degree.

Factor analysis supported the five‐dimensional structure of the modified inventories and all scales demonstrated good reliability (Table 1).^31^

There were statistically significant differences between patients and dietitians on each scale in the inventory, except for “patient‐dietitian communication” (Table 2). Patients reported significantly higher median scores compared to dietitians for “shared decision‐making,” but significantly lower scores for “providing holistic and individualized care,” “knowing the patient/dietitian” and “caring patient‐dietitian relationships” (Table 2). Despite these statistically significant differences, scores were relatively high across all scales for both groups.

**Table 2:** Results of Mann‐Whitney *U* test comparing total scores for all 5 scales between patients (n = 133) and dietitians (n = 180)

PCC Dimension	No. scale items	Possible range	Median (IQR) score		*U*	*P* (two‐tailed)
			Patient (N = 133)	Dietitian (N = 180)		
Patient‐dietitian communication	7	7‐35	35.0 (5)	33.0 (4)	10 480	0.130*
Shared decision‐making in dietetics	8	0‐40	33.0 (14)	32.0 (7)	9233	**0.004**
Holistic and individualized dietetic care	8	8‐40	36.0 (8)	38.5 (5)	9464	**0.005**
Knowing the patient/dietitian	3	0‐12	8.0 (5)	9.0 (2)	9189	**0.001**
Caring patient‐dietitian relationships	5	0‐12	11.0 (3)	11.0 (2)	9923	**0.009**

IQR, interquartile range; PCC, patient‐centred care; *U*, Mann‐Whitney *U* statistic.

Significant *P*‐values (<0.05) are bolded.

* *P*‐value = 0.041 when outliers were removed.

## 4 DISCUSSION

To our knowledge, this study is the first to compare patients’ and dietitians’ perceptions of PCC in dietetic practice. We have gained an initial understanding of differences between patients’ and dietitians’ perceptions. These findings suggest that work is needed to address these discrepancies to ensure care is aligned with patients’ needs and expectations.

It is important to note that the Mann‐Whitney *U* test can detect differences in spread even when the differences in median values are small.^35^ This may have contributed to the significant differences observed between dietitians’ and patients’ median scores despite scores being relatively high overall. The spread of scores for patients was greater than for dietitians. Thus, patients may have been a less homogenous group compared to dietitians regarding their perceptions of PCC in dietetic consultations.

There are several factors that may have influenced the greater variability observed among patients’ scores. For example, there may have been differences between dietetic practices relating to the model of care, dietitians’ caseload and continuity of patient‐dietitian assignment (eg, one‐third of patients reported not having a regular dietitian). It is also possible that patients’ characteristics (such as age, gender and reason for visiting the dietitian) influenced the variability in patients’ responses, though we were unable to control for these factors in our analyses. Further, patients’ preferences for and perceived relevance of specific aspects of PCC may have influenced their interpretation of items. It may be beneficial for future research to explore some of these factors further.

Patients’ rated dietitians significantly lower for providing holistic and individualized care. Items on this scale relate to skills/behaviours such as treating patients uniquely, tailoring care specifically to patients’ needs and taking the time to find out more about the patient as a person. This finding shares similarities with a previous qualitative study conducted in Australia involving semi‐structured interviews with 11 patients who had attended consultations with dietitians working in primary care.^36^ Participants emphasized the need for dietitians to explore and understand the unique factors influencing patients’ health and illness (eg, living situation, budget) and some patients explained that non‐individualized resources and strategies were unhelpful and unrealistic.^36^ Further, in a cross‐sectional study conducted in the UK, only 11% of dietitians reported considering the extent to which written information accounted for patients’ individual needs.^37^ Providing individualized care is important to patients and is also a key component in professional standards for dietitians.^38^, ^39^, ^40^ To provide a positive and helpful experience for patients, it is important to address differences in patients’ and dietitians’ perceptions of the provision of holistic and individualized care.

Both patients’ and dietitians’ scores were not particularly high for “knowing the patient/dietitian.” Items on this scale related to patients and dietitians knowing one another, dietitians knowing each patient as a person, and understanding how their patients feel. It is possible that continuity of patient‐dietitian assignments may have influenced the lower scores for this dimension of PCC; one‐third of patients reported not having a regular dietitian, a factor that is likely to influence the extent to which patients and dietitians know and understand one another. The patient‐health professional relationship is an integral component of PCC.^5^ Further, the importance of positive patient‐dietitian relationships has been emphasized by patients^36^, ^41^ and referred to by international dietetic professional standards.^38^, ^39^, ^40^ Clearly, it is important to consider strategies for dietitians to foster and maintain good relationships with patients, including considering strategies that might work when time is limited, and patients are unlikely to regularly visit the dietitian. If good relationships are developed, this may encourage patients to engage in on‐going care with their dietitian.

Patients rated dietitians significantly higher regarding shared decision‐making compared to dietitians’ self‐ratings. Aspects of shared decision‐making captured by the 9‐item Shared Decision‐Making Questionnaire (SDM‐Q‐Doc/SDM‐Q‐9) include the following: dietitians and patients discussing the advantages and disadvantages of different nutrition care options; dietitians helping patients understand the information and eliciting patients’ preferences for the different options; and dietitians and patients selecting nutrition care options together. This finding is unexpected given evidence from previous studies. For example, two previous observational studies found the level of shared decision‐making in dietetic consultations to be quite low; shared decision‐making was assessed with the OPTION scale (observing patient involvement in decision making), and the mean OPTION scores were 28%^42^ and 29%^43^ out of a possible 100% (ie, 0% = no patient involvement in the decision; 100% = high patient involvement). Further, findings from a recent qualitative study indicate that patients sometimes perceive dietitians as being dictatorial and controlling the encounter, inhibiting patients’ participation in decision‐making.^36^ It is important to note that patients have traditionally been “passive” recipients of care, with healthcare professionals assuming the “expert” role.^44^ Therefore, if patients have limited experience with being actively involved in care, their expectations regarding their level of involvement may be low,^44^ and this may partly explain patients’ high overall ratings in this study. However, it is also possible that the group of dietitians whom patients were referring to were particularly skilled in shared decision‐making practices, and/or that patients’ subjective ratings of dietitians shared decision‐making behaviours were more “generous” than observational data.

There are several factors that may influence dietitians’ perceptions of their shared decision‐making practices. In a qualitative study exploring dietitians’ salient beliefs regarding shared decision‐making, dietitians perceived that time constraints, disapproval from physicians and patient factors (eg, patients’ personalities, motivation, level of understanding) were barriers to engaging patients in shared decision‐making.^45^ These barriers were also identified in an observational study using the OPTION scale; dietitians’ likelihood of adopting shared decision‐making was influenced by the patient's health condition, lifestyle habits and having insufficient time.^42^ It is also possible that dietitians’ attitudes towards shared decision‐making influenced their responses; dietitians have previously described potential disadvantages of presenting options to patients, such as making patients feel less secure and increasing dietitians’ feelings of incompetence.^45^ Further, dietitians do not always agree with the importance of recognizing patients’ as experts in their own nutrition care.^46^

It is possible that discrepancies between patients’ and dietitians’ perceptions were influenced by differences in the group of dietitians who responded to the survey, and the group of dietitians whom patients were evaluating. We recommend that future research sample dyads of patients and dietitians whereby they both complete the survey individually following the consultation. This would allow individual dietitians to better understand their own strengths and weaknesses regarding their PCC practices. It could also help dietitians to develop their own practice improvement goals and establish practical strategies, perhaps with input from mentors/peers, as to how they can advance their skills in specific areas. If this was performed across several sites/dietetic practices, it would also be possible to combine the results to highlight areas that are consistent “weaknesses” across different practices and signal opportunities for group education/quality improvement.

## 5 STRENGTHS AND LIMITATIONS

A strength of this study is the use of valid and reliable patient‐ and dietitian‐reported inventories. Further, the anonymous, self‐administered nature of the survey was designed to reduce response bias and maximize honesty, and both surveys were able to be completed at a time most convenient to participants, without the influence of others. To reduce the risk of dietitians distributing patient surveys selectively (eg, giving surveys to patients who would provide a desirable assessment), dietitians were instructed to invite consecutive patients, and it was clearly communicated to practices that individual dietitians would not be identifiable. However, we were unable to directly prevent or monitor this and therefore cannot say it did not occur.

These findings may not be generalizable to a larger population given the small sample size and response rate of only 30% for patients and low estimated response rate by dietitians; we were unable to determine an exact response rate for dietitians. Further, we do not know the characteristics of patients who did not complete the survey, nor their reason for not responding. Finally, there may be other factors that influenced patients and dietitians scores that we were unable to control for in the present study (eg, the patient's reason for attending the consultation and their health status, dietitians recent experience prior to completing the survey, and their practice situation).

It is important to note that while patients were asked to rate the dietetic consultation in which they received the survey, dietitians rated their delivery of care in general and therefore may have been referring to any number of consultation experiences. This is not a direct comparison; patients may be referring to dietitians other than those who responded to the survey (ie, the sample of dietitians may or may not include the pool of dietitians whom recruited patients/patients were reflecting on).

Finally, due to the violation of assumptions (eg, non‐normality of continuous data, even after data transformation), we were unable to employ a robust method such as regression analysis to explore characteristics that might influence participants’ scores. This may be an important gap for future research to address.

## 6 PRACTICE/RESEARCH IMPLICATIONS

It is important that dietitians are made aware of these survey results along with suggestions on how to ensure patients’ and dietitians’ perceptions and experiences are aligned. Communicating these findings may stimulate dietitians’ self‐reflection and awareness regarding their PCC practices, providing an initial step to bridge any gaps between dietitians’ and patients’ perceptions. After all, a patient‐centred healthcare professional should be self‐reflective.^5^ These findings could be incorporated into workshops and online webinars for continued professional development, including audio or video learning tools; and in presentations/in services, particularly to those practices who participated in this study.

There is potential for the inventory to be used as a tool to stimulate dialogue between patients and dietitians regarding their perceptions of and preferences for PCC. For example, the inventory could be modified and used as a “checklist” of actions/behaviours. Dietitians and patients could then consider and discuss the importance of these actions/behaviours at the beginning of the consultation. This would provide dietitians and patients with a better understanding of one another's needs and preferences.

Establishing a shared understanding at the beginning of the consultation may help foster positive relationships and collaboration between patients and dietitians. Further, providing patients with a tool that helps them articulate what is important to them gives patients an active role in ensuring their care is patient‐centred, rather than dietitians being solely responsible for “fixing” or “improving” their practices. After all, PCC is about ensuring patients are actively engaged in their care.

While the inventory may have great practical value, additional research is needed. The validity of the inventory needs further evaluation. More work may also be needed to establish benchmarks/minimally acceptable scores; what score signifies need for improvement; and the parameters for what is an acceptable score. Further, while the inventory allows patients and dietitians to report on the extent to which they experience PCC, it does not gauge how important these aspects of PCC are to respondents. Therefore, a valuable addition to the instrument may be inclusion of a section that allows respondents to describe or rate the importance of the different aspects. Understanding the value patients and dietitians place on PCC would give depth and context to these findings. Further, dietitians’ and patients’ perceptions regarding the effectiveness of the inventory as a learning and evaluation tool should be explored; while pilot interviews were conducted during the development phase,^23^, ^24^ it may be necessary to evaluate the importance/usefulness of the inventory among a larger sample.

## 7 CONCLUSION

This study uncovered two key findings. Firstly, the results highlighted potentially important differences between patients’ and dietitians’ perceptions of PCC. Further, greater spread of scores for patients compared to dietitians may be indicative of patients varying preferences; it is important to check patients’ preferences and tailor care accordingly. Secondly, this study identified aspects of dietetic care that may require practice improvements. In particular, patients’ ratings were significantly lower compared to dietitians for “providing holistic and individualized care,” “knowing the patient/dietitian” and “caring patient‐dietitian relationships.” Finally, these findings may promote self‐reflection and awareness regarding PCC practices, and provide an initial step to bridge gaps between dietitians’ and patients’ perceptions.

## CONFLICT OF INTEREST

None.

## ETHICAL APPROVAL

The approval was obtained from the institution's Human Research Ethics Committee (REF: 2017/730). Participation was voluntary and only non‐identifiable data were collected. No incentive was provided.
